# Acute Mountain Sickness Symptom Severity at the South Pole: The Influence of Self-Selected Prophylaxis with Acetazolamide

**DOI:** 10.1371/journal.pone.0148206

**Published:** 2016-02-05

**Authors:** Michael F. Harrison, Paul J. Anderson, Jacob B. Johnson, Maile Richert, Andrew D. Miller, Bruce D. Johnson

**Affiliations:** 1 Division of Cardiovascular Diseases, Mayo Clinic, Rochester, Minnesota, United States of America; 2 Department of Emergency Medicine, Henry Ford Hospital, Detroit, Michigan, United States of America; 3 Division of Preventive, Occupational, and Aerospace Medicine, Mayo Clinic, Rochester, Minnesota, United States of America; University of California Los Angeles, UNITED STATES

## Abstract

**Introduction:**

Acetazolamide, a carbonic anhydrase inhibitor, remains the only FDA approved pharmaceutical prophylaxis for acute mountain sickness (AMS) though its effectiveness after rapid transport in real world conditions is less clear.

**Methods:**

Over 2 years, 248 healthy adults traveled by airplane from sea level (SL) to the South Pole (ALT, ~3200m) and 226 participants provided Lake Louise Symptom Scores (LLSS) on a daily basis for 1 week; vital signs, blood samples, and urine samples were collected at SL and at ALT. Acetazolamide was available to any participant desiring prophylaxis. Comparisons were made between the acetazolamide with AMS (ACZ/AMS) (n = 42), acetazolamide without AMS (ACZ/No AMS)(n = 49), no acetazolamide with AMS (No ACZ/AMS) (n = 56), and the no acetazolamide without AMS (No ACZ/No AMS) (n = 79) groups. Statistical analysis included Chi-squared and one-way ANOVA with Bonferroni post-hoc tests. Significance was p≤0.05.

**Results:**

No significant differences were found for between-group characteristics or incidence of AMS between ACZ and No ACZ groups. ACZ/AMS reported greater LLSS, BMI, and red cell distribution width. ACZ/No AMS had the highest oxygen saturation (O_2_Sat) at ALT. No significant differences were found in serum electrolyte concentrations or PFT results.

**Discussion:**

Acetazolamide during rapid ascent provided no apparent protection from AMS based on LLSS. However, it is unclear if this lack of effect was directly associated with the drug or if perhaps there was some selection bias with individuals taking ACZ more likely to have symptoms or if there may have been more of perceptual phenomenon related to a constellation of side effects.

## Introduction

Travel to altitude higher than 2500m is associated with a risk of developing acute mountain sickness (AMS) [1–3], the least serious of the altitude related illnesses that also include high-altitude pulmonary edema (HAPE) and high-altitude cerebral edema (HACE). The development of AMS has not been clearly associated with any singular factor nor has a definitive etiology of AMS been identified [1–3]. Rather many factors such as age, gender, body habitus, physical fitness, tolerance of hypocapnia, rate of ascent, exertion level, previous problems at altitude, recent prior ascents or individual susceptibility have been linked to increased risk of AMS but conflicting reports raise questions about the importance or contribution of each of these variables [1,3,4].

Ascending slowly to permit acclimatization is an effective means of AMS prevention [4] but pharmacological options are available for those individuals who are unable or unwilling to perform a slow ascent. Prophylactic options include carbonic anhydrase inhibitors with diuretic effects such as acetazolamide [2,5,6] or low-dose corticosteroids such as dexamethasone [5,7,8]. Acetazolamide is the FDA approved gold standard for pharmaceutical prophylaxis and multiple studies suggest it is 60–80% effective at mitigating AMS [6,9]. The Wilderness Medical Society gives acetazolamide a grade 1C recommendation for prevention and treatment of AMS [5]. Other recent publications, however, suggest that non-steroidal anti-inflammatory (NSAID) medications such as ibuprofen are just as effective [10,11], despite only receiving a grade 2B recommendation from the Wilderness Medical Society [5]. NSAIDs and corticosteroids are hypothesized to decrease the risk of AMS development by addressing the inflammatory response that is associated with hypoxic environments [10,11].

How acetazolamide prevents AMS is not completely understood [2,6]. Most proposals suggest that acetazolamide inhibits renal re-absorption of bicarbonate and produces a metabolic acidosis that is compensated by an increased respiratory rate and improved oxygenation [12,13]. However, one review posits this conventional explanation is too simplistic [2]. Other mechanisms of action include decreased cerebrospinal fluid production, improved arterial oxygen saturation and prevention of further impairment of gas exchange, and improved sleep quality [2,10, 14]. Swenson & Teppema [13] summarize acetazolamide’s effects on stimulating the hypoxic ventilatory response but they also highlight some of the unanswered questions related to the protective effects of acetazolamide with respect to AMS. In all of this literature, however, it is unclear if acetazolamide would provide prophylactic effects in field conditions that involve self-administered medications, unpredictable travel schedules, and rapid transport to high altitude.

A previous publication has summarized the incidence and symptoms of AMS in Antarctic workers [15]. However, it did not comment on the underlying physiologic changes associated with passive transportation of healthy adults to altitude at the South Pole nor did it assess sub-group populations stratified by acetazolamide use and acute mountain sickness symptom scores. Our primary goal was to investigate the field effectiveness of acetazolamide in a healthy adult population who used acetazolamide prophylaxis at their own discretion prior to rapid transport to high altitude. While our previous publication indicated similar rates of AMS amongst those who did and who did not opt to use acetazolamide prophylactically in the field, we hypothesized that those who did opt to use acetazolamide prophylactically would have less severe symptoms. Our secondary goal was to compare anthropometric and physiologic differences (e.g. oxygenation, plasma volume, urine specific gravity) based on acetazolamide use to further investigate mechanism(s) by which acetazolamide mitigates AMS.

## Methods

Data were collected during two austral summer expeditions to the Amundsen-Scott South Pole Station. Ethical approval was obtained from Mayo Clinic, Rochester MN and all participants provided written informed consent. Participants were included in the study if their duties at Amundsen-Scott South Pole Station exceeded one week in duration. During 2006–2007, data were only collected from those who had not been a participant during the 2005–2006 expedition.

Typically, participants remained at the sea level McMurdo Station for ~1–2 weeks. Subsequently the majority of participants flew to the South Pole in an airplane that was pressurized after take-off but depressurized during the flight so that cabin pressure had equilibrated with ambient atmospheric pressure at the time of landing. During the initial time at sea level, participants underwent baseline testing and education related to high altitude illness. Acetazolamide was made available to any participant who wished to employ AMS prophylaxis. Acetazolamide was distributed in packets that instructed dosing at 250mg by mouth twice daily, starting 24–48 hours prior to anticipated travel to the Amundsen-Scott South Pole Station. Questionnaires collected at baseline included Lake Louise Scores for AMS symptoms as well as information related to past medical history and chronic medical conditions, current medication use, lifestyle assessment (i.e. tobacco and alcohol use; exercise habits), and previous experience with altitude and/or Antarctic expeditions. Baseline anthropometric and physiological measurements included height, weight, heart rate, blood pressure, arterial oxygen saturation (SaO_2_) and a blood draw. Blood draws were performed at to sea-level ~1–2 days prior to departure to altitude. A repeat blood draw was performed on the third day after arrival at altitude. Blood samples were analyzed for hemoglobin concentration and hematocrit; serum electrolyte and progesterone levels; circulating catecholamine levels; and thyroid, liver and kidney function. Changes in plasma volume were calculated using Dill and Costill’s method [16].

Participants completed the same questionnaire reporting AMS symptoms on 9 separate occasions. Questionnaires were completed at baseline, on the plane to the Amundsen-Scott South Pole Station, and daily for the first seven days following arrival. Completion of the first questionnaire at the Amundsen-Scott South Pole Station occurred prior to sleep on the first night and each of the subsequent questionnaires were completed upon waking. An individual was determined to be suffering from AMS if their Lake Louise Symptom Score was ≥ 3 concurrent with a headache at any time during the first 7 days at altitude. As per our prior publication, symptoms peaked within 72 hours for the majority of participants [15].

Statistical analyses were performed with SPSS 22.0 (IBM Corporation, Armonk NY). Chi-square test was performed to analyze rates of AMS occurrence between acetazolamide users and non-users. A Mann-Whitney U test completed the non-parametric analysis of the other data. A t-test was used to evaluate the difference between the relative acetazolamide dose (mg/kg/day) for the two groups that were taking it. Significance was set as p≤0.05.

## Results

Over the 2 austral summer periods, 248 individuals provided informed consent and 22 participants were excluded for incomplete questionnaire data related to personal history or AMS symptoms (n = 14) or hematologic samples not suited for analysis due to hemolysis (n = 8). Characteristics of the 226 participants included in the final analysis are presented in Table 1. Biochemical results are in Table 2 while hematological results are present in Table 3. No significant differences were noted for any of the pulmonary function test results based on subgroup stratification and the results for the entire population has been published previously [**17**]. No differences were noted between subpopulations for any of the endocrinologic or adrenergic results including progesterone, epinephrine, norepinephrine, dopamine, TNF-α, or VEGF. These are not presented in the interest of brevity.

**Table 1:** Subject Characteristics.

Variable	No Acetazolamide		Acetazolamide		F-score / T-	p-value
	No AMS	AMS	No AMS	AMS	score	
N (%)	79 (35)	56 (25)	49 (22)	42 (18)	n/a	0.11
Male N (%)	59 (75)	36 (64)	34 (69)	29 (53)	n/a	0.10
Age (yrs)	36.7 ± 9.9	35.2 ± 9.5	39.4 ± 11.6	39.5 ± 11.4	1.95	0.12
Experience >3000m N (%)	70 (89)	53 (95)* Ω	40 (82)*	35 (83) Ω	n/a	0.05
Prior AMS N (%)	34 (43)	33 (58)	29 (59)	27 (64)	n/a	0.12
Height (m)	1.77 ± 0.09	1.74 ± 0.08	1.74 ± 0.09	1.77 ± 0.09	1.68	0.17
Wt (kg)	82.9 ± 14.3*	74.9 ± 13.3*Ω	79.4 ± 14.9	85.7 ± 17.1 Ω	5.44	<0.01
BMI (kg/m^2^)	26.5 ± 3.6	24.6 ± 3.5*	26.0 ± 4.2	27.4 ± 5.2*	4.21	0.01
Acetazolamide Dose (mg/kg/day)	n/a	n/a	6.8 ± 1.0*	6.1 ± 1.2*	5.481	<0.01
Neck Circumference (cm)	36.9 ± 3.7*	36.3 ± 3.2	35.4 ± 3.1*Ω	37.3 ± 3.9Ω	3.36	0.02
Residence Elevation (m)	3402.0 ± 3001.0	2680.0 ± 2651.5	2104.7 ± 2392.5	2722.5 ± 2599.4	2.34	0.07
Max LLSS	1.2 ± 0.8*Ω	3.1 ± 1.5*ϕε	1.1 ± 0.7ϕγ	4.0 ± 2.0Ωγε	65.80	<0.01
Heart Rate at Sea Level (beats/min)	70.7 ± 12.6	71.9 ± 11.9	73.0 ± 12.8	70.8 ± 12.4	0.40	0.75
Heart Rate at South Pole (beats/min)	80.8 ± 14.1	84.7 ± 12.8	82.3 ± 12.9	84.9 ± 12.1	1.21	0.31
Systolic Blood Pressure at Sea Level (mmHg)	112.1 ± 13.6	109.4 ± 12.0	111.9 ± 11.1	111.1 ± 12.4	0.61	0.61
Diastolic Blood Pressure at Sea Level (mmHg)	71.2 ± 9.9	67.2 ± 9.2	69.7 ± 9.3	70.5 ± 9.9	2.04	0.11
Systolic Blood Pressure at South Pole (mmHg)	108.6 ± 16.0	105.3 ± 15.3	103.3 ± 13.3	103.9 ± 13.0	1.48	0.22
Diastolic Blood Pressure at South Pole (mmHg)	69.8 ± 9.6	67.2 ± 10.1	68.1 ± 9.8	68.3 ± 9.6	0.75	0.52
O2Sat at South Pole (%)	89.6 ± 2.6*	89.9 ± 3.6	91.2 ± 1.8*ϕ	90.0 ± 2.9ϕ	3.89	0.01

*, ε, γ, ϕ, Ω—indicates significant differences, p<0.05; Degrees of freedom = 3

**Table 2:** Biochemical Variables.

Variable	No Acetazolamide		Acetazolamide		F-score	p-value
	No AMS	AMS	No AMS	AMS		
Sodium (mEq/L)	139.2 ± 2.5	138.8 ±1.6	139.2 ± 2.0	139.3 ± 2.1	0.62	0.60
Potassium (mEq/L)	4.2 ± 0.4	4.3 ± 0.6	4.2 ± 0.3	4.2 ± 0.3	0.46	0.71
Chloride (mEq/L)	102.3 ± 3.0	102.0 ± 2.4	102.8 ± 2.6	102.7 ± 3.3	0.75	0.52
Creatinine (mg/dL)	1.0 ± 0.1	1.0 ± 0.1	1.0 ± 0.2	1.0 ± 0.1	2.53	0.06
Urine pH	6.37 ± 0.85	6.53 ± 0.74	6.38 ± 0.68	6.32 ± 0.71	0.67	0.57
Urine Specific Gravity	1.02 ± 0.01	1.02 ± 0.01	1.02 ± 0.01	1.02 ± 0.01	0.04	0.99
Glucose (mg/dL)	87.1 ± 10.1	84.7 ± 7.5	89.8 ± 11.9	89.0 ± 11.1	2.52	0.06
Calcium (mEq/L)	9.6 ± 0.4	9.6 ± 0.4	9.6 ± 0.4	9.6 ± 0.3	0.49	0.69
AST (U/L)	23.8 ± 9.7	21.3 ± 5.3	24.4 ± 8.8	24.8 ± 14.3	1.44	0.23
ALT (U/L)	24.5 ± 14.4	20.2 ± 11.9	27.2 ± 15.0	25.5 ± 15.4	2.34	0.07
Alkaline Phosphatase (U/L)	68.5 ± 17.1	66.8 ± 25.0	69.4 ± 18.9	69.1 ± 18.3	0.18	0.91
Total Cholesterol (mg/dL)	183.1 ± 35.3	184.5 ± 31.5	188.8 ± 32.8	186.4 ±30.6	0.32	0.81
LDL (mg/dL)	104.2 ± 30.0	100.4 ± 25.7	107.2 ± 31.7	110.3 ± 30.8	1.02	0.39
HDL (mg/dL)	57.3 ± 14.0	64.4 ±17.0*	59.6 ± 15.6	54.7 ± 17.0*	3.56	0.02
VLDL (mg/dL)	21.3 ± 13.0	19.7 ± 9.8	20.5 ± 10.1	21.7 ± 10.9	0.31	0.82
Triglycerides (mg/dL)	109.6 ± 72.2	100.4 ± 52.1	110.4 ± 58.0	106.8 ± 53.0	0.32	0.81
Chol: HDL Ratio	3.3 ± 1.0	3.0 ± 0.8*	3.3 ± 0.9	3.7 ± 1.1*	3.89	0.01

*—indicates significant differences, p<0.05; Degrees of freedom = 3

**Table 3:** Hematological Variables.

Variable	No Acetazolamide		Acetazolamide		F-score	p-value
	No AMS	AMS	No AMS	AMS		
WBC (10^9^/L)	6.0 ± 1.6	5.9 ± 1.7	6.0 ± 1.6	5.9 ± 1.8	0.08	0.97
RBC (10^9^/L)	5.0 ± 0.4*	4.8 ± 0.4*	4.9 ± 0.5	4.9 ± 0.4	2.79	0.04
Hemoglobin (g/dL)	15.4 ± 1.2	15.0 ± 1.0	15.2 ± 1.3	15.3 ± 1.4	1.52	0.21
Hematocrit (%)	45.5 ± 3.4	44.4 ± 3.0	45.2 ± 3.8	45.5 ± 3.8	1.28	0.28
Platelet (10^9^/L)	226. 8 ± 46.1*	253.4 ± 54.5*	241.9 ± 52.6	249.6 ± 67.9	3.13	0.03
MCV (μg/cell)	91.8 ± 4.1	93.5 ± 3.8	93.2 ± 5.1	92.3 ± 5.4	1.84	0.14
MCH (μg/cell)	31.0 ± 1.3	31.5 ± 0.9	31.5 ± 1.2	30.9 ± 1.6	2.76	0.04
MCHC (μg/cell)	33.8 ± 0.8	33.7 ± 0.9	33.6 ± 1.1	33.5 ± 1.1	0.93	0.43
RDW (%)	13.7 ± 1.0*	13.3 ± 0.8Ω	13.6 ± 0.9	14.2 ± 1.5*Ω	6.25	<0.01
Eosinophils (10^9^/L)	2.2 ± 1.8	2.5 ± 1.9	2.3 ± 1.9	2.4 ± 2.3	0.22	0.89
Estimated Change in Plasma Volume (%)	-7.2 ± 11.1	-7.5 ± 11.9	-4.6 ± 13.2	-6.1 ± 13.3	0.51	0.67
Iron (μg/dL)	112.5 ± 38.8	127.3 ± 51.8	122.4 ± 41.2	109.5 ± 45.9	1.93	0.13
Iron Saturation (%)	35.9 ± 14.0	39.6 ± 14.9	39.8 ±15.8	34.6 ± 15.7	1.57	0.20
TIBC (μg/dL)	323.0 ± 45.1	321.6 ± 40.6	316.9 ± 52.4	326.6 ± 49.1	0.34	0.79
UIBC (μg/dL)	210.2 ± 59.4	195.9 ± 55.3	195.3 ± 65.5	216.0 ± 76.2	1.23	0.30

*, Ω—indicates significant differences, p<0.05; Degrees of freedom = 3

## Discussion

Our data revealed that acetazolamide was associated with more severe subjective symptom scores. In our field study, 46% (42/91) of those using acetazolamide reported symptoms consistent with AMS and the ACZ/AMS group reported increased symptom severity compared to the AMS/No ACZ group (4.0 ± 2.0 and 3.1 ± 1.5, p<0.01). A detailed summary of symptom patterns in our participant population has been presented previously [15]. In controlled settings, acetazolamide use is reported to be 60–80% effective in preventing AMS [6] whereas we report decreased effectiveness of acetazolamide in a large field expedition under real world conditions. The possible explanations for this unexpected finding are likely related to the mild nature of AMS in our population, the subjective nature of the diagnosis of AMS, and the side effect profile of acetazolamide. Our participants largely reported symptom scores that correlate with mild AMS and, in a significantly smaller sample population (N = 15), acetazolamide was demonstrated to have minimal impact on LLSS when the LLSS is <7 [18]. Acetazolamide’s possible side effects include headache, dizziness, dyspnea, asthenia, nausea, vomiting, loss of appetite, and gastrointestinal discomfort [4,6]. These symptoms closely mirror AMS symptoms, the condition our participants would have used acetazolamide to prevent and the true prevalence of adverse events from acetazolamide use at high altitude is not often well reported [19,20]. Given the subjective nature of the diagnosis of AMS, those in the ACZ/AMS may have rated their symptoms as more severe based on the expectation that acetazolamide would render them symptom-free during the expedition. It may have been even more difficult to ascertain the true cause of the constellation of symptoms using a self reported tool such as the Lake Louise Symptom Score rather than one of the other tools, such as Hackett’s established AMS score [21], that requires an examination by a medical professional. However, with our large sample population, the frequent evaluations during transport and the first week of altitude exposure, and the austere conditions with limited medical resources at the South Pole, the use of another tool would not have been feasible. Future work should consider the utilization of another tool that would include a medical exam because at present, it is difficult to determine if the symptoms reported by our participants causing our unexpected results were AMS symptoms, side effects from acetazolamide, or a psychological phenomenon.

Our results are based on the principle of “intention to treat” with our participants selecting to use or not to use acetazolamide based on a variety of personal factors (i.e. prior AMS, prior high altitude experience, anxiety about AMS etc). Each participant was responsible for their own dosing schedule once therapy was initiated. Additionally, the timing of the initiation of therapy prior to travel to high altitude may have also contributed to our findings. Where our participants started their regimen approximately 24–48 hours prior to their anticipated travel, a recent publication with a small sample size suggests that a minimum of 48 hours of treatment prior to arrival at high altitude is associated with decreased symptom score during passive transport by car or cable car from an initial altitude of 600m [22]. Perhaps for rapid travel from sea level to high altitude, acetazolamide treatment should be initiated 48–72 hours prior to travel. Regardless, very few differences are reported in subject characteristics (Table 1) and the large sample population makes it likely that any confounding effects would be evenly distributed between the four subpopulations.

The available literature related to acetazolamide dosing warrants discussion. A trio of meta-analyses support an acetazolamide dose of 250mg twice daily as the minimum effective dose as compared to placebo to prevent AMS [19,20,23]. While acetazolamide 250mg twice daily is a commonly recommended dose and schedule [5], perhaps it is insufficient for rapid travel to high altitude. Carbonic anhydrase, the enzyme inhibited by acetazolamide, exists in a number of tissues and structures including the collecting tubules of the kidneys, red blood cells, the ciliary processes of the eyes, and the epithelial cells of the choroid plexus. Due to pharmokinetics, doses of 3-5mg/kg/day are reported to be insufficient for benefits related to AMS [2]. An acetazolamide dose of >5mg/kg/day is sufficient to have effects on the kidneys and the red blood cells but increased doses approaching 10mg/kg/day may be required in order to affect other locations such as red blood cells and 20mg/kg/day may be required to penetrate the blood brain barrier and effect the choroid plexus to decrease cerebrospinal fluid (CSF) production [2,6,7]. The ACZ/No AMS group had a significantly larger relative dose as compared to the ACZ/AMS group. While neither group would be described as subtherapeutic by Leaf & Goldfarb’s [2] review, perhaps the ACZ/AMS group members were not experiencing the full central nervous system benefits and hence the increased symptom score. The role of cerebral edema in AMS has been debated with no clear consensus [4] though hypoxia can cause elevated intracranial volume and pressure [6]. The inclusion of “headache” as a cardinal symptom in the diagnosis of AMS by LLSS suggests a role for perhaps subclinical cerebral edema [24] and mild intracellular cerebral edema has been demonstrated on advanced imaging with smaller sample populations [25]. Using a research methodology that is not likely to be repeated in the near future, Wilson et al [26] demonstrated that intracranial pressure increased with even the slightest exertion at altitude in an individual diagnosed with AMS while an inverse correlation between ventricular size and headache score was reported. Teppema et al [27] provide evidence that acetazolamide use may reduce cerebral edema but used a dose of 250mg every 8h, a 50% increase in dose as compared to our methodology.

With respect to our secondary aim of identifying anthropometric and physiologic differences that may be related to the proposed mechanism by which acetazolamide may prevent AMS development, our current results demonstrate some interesting findings. Acetazolamide decreases both the occurrence and severity of apneic periods and is associated with improved oxygen saturation [14,28,29]. The ACZ/No AMS group had the greatest oxygen saturation at altitude as measured with pulse oximetry though no differences were observed in either urine pH or urine specific gravity that would support either an increased diuretic effect or provide evidence of urinary acid-base changes suggestive of metabolic acidosis. The ACZ/AMS group had the greatest body mass, greatest BMI, the greatest neck circumference, and greatest RDW. These variables are factors associated with the diagnosis of sleep apnea and sleep apnea severity [30]. For these individuals, this may have had a deleterious effect at altitude as apneic periods occur with increased frequency at high altitude, result in poor sleep quality, and are hypothesized to contribute to the development of AMS [31,32]. Only 2 of our subjects carried the diagnosis of OSA and neither were in the ACZ/AMS group (both were in the AMS/No ACZ group).

These discussion points raise important points for selecting the most appropriate pharmaceutical agent for AMS prophylaxis when expecting rapid travel to altitude. While acetazolamide has performed well in randomized controlled trials for either prophylaxis [33] or treatment [14] of AMS, our study highlights some performance concerns under real-world conditions in a large field expedition. Our results did not reproduce the findings of the rigorously controlled studies. The significance of this finding to multiple populations (i.e. workers travelling to the South Pole, military personnel preparing for rapid deployment to high altitude, or the general public travelling to high altitude for vacation) is even more important given the current recommendations summarized in one source as “a dose of 125 mg to 250 mg acetazolamide taken twice daily begun the day before arrival at high altitude”[22]. Our results indicate this approach may not always work under these field conditions. With respect to what alternatives remain for pharmaceutical prophylaxis for individuals who are rapidly transported to altitude, more research is required. According to the most recent guidelines, acetazolamide only receives a level 1C grade (strong recommendation, low-quality or very low-quality evidence) [5]. Dexamethasone receives the highest grade at 1A (strong recommendation, high-quality evidence) with benefit for specific situations similar to what our methodology entailed with respect to rapid and passive transport via airplane [5] and one study suggests a synergistic interaction between acetazolamide and dexamethasone as compared to acetazolamide monotherapy [9]. NSAIDs receive a 2B grade (weak recommendation, moderate-quality evidence) [5] and their side effect profile of in comparison to glucocorticoids makes them an attractive target for future research into AMS prevention. Based on the available references related to NSAID use and AMS prevention [10,11], it is likely that the lower score for NSAIDs is influenced by its novelty and future work may influence a stronger recommendation in the future if the current results are consistently reproduced. Our recent publication lends further support to the role of inflammation in the development of AMS amongst a population of healthy adults not employing acetazolamide while experiencing rapid ascent to altitude [34].

Our study has a number of strengths. We present a large cohort of healthy adults who were uniformly transported to an altitude at which AMS is likely to occur in a uniform matter that was both rapid and passive. We collected a comprehensive data set that included detailed questionnaire data, vital signs and anthropometrics, blood and urine samples, and pulmonary function tests. This consistency is important as study design has been linked to reported incidence rates of AMS [35]. The most obvious limitation of our study is that acetazolamide was mandated to be available to any participant who wished to take it–this was a potential source of bias and made a controlled study impossible. However, no differences were observed in relation to which subjects self-selected to use acetazolamide based on prior AMS or altitude exposure. As a result, it appears that the effects of this on our results may have been small. Furthermore, the application of an intention to treat analysis is comparable to what would be realistically employed by the general public based on the readily available recommendations by authoritative bodies such as the Wilderness Medical Society [5]. Regardless of the exact cause of our results–legitimate mild-to-moderate AMS, a medication side effect, a psychological phenomenon, or a combination thereof–the more severe constellation of symptoms subjectively reported by those taking acetazolamide has potential to disrupt occupational (e.g South Pole and military operations) and recreational (e.g. vacation travel to mountain resorts) activities of an individual who travels directly to high altitude in a rapid manner and warrants further investigation.

## Conclusion

In a population of healthy adults who were transported to high altitude in a rapid and passive manner, the incidence of AMS was unexpectedly similar between individuals taking acetazolamide and those that did not. Individuals who developed AMS despite taking acetazolamide reported more severe symptoms as compared to the other groups. We conclude that acetazolamide may actually intensify symptom reporting in individuals who take prophylaxis and go on to develop AMS and that this is primarily due to subjective nature of AMS diagnosis, variability in self-administration of medications, medication side effects, and participant expectations. These uncontrolled variables may also contribute to a decreased prophylactic effectiveness of acetazolamide in field trials. These findings generate questions that should motivate further investigations into guidelines about the use of acetazolamide in the field when rapid transport is anticipated and travel schedules are uncontrollable for recreational or occupational purposes.

## Supporting Information

S1 Data Excel spreadsheet of raw data used for the present analysis. (XLSX)
